# Deep mutational analysis of elongation factor eEF2 residues implicated in human disease to identify functionally important contacts with the ribosome

**DOI:** 10.1016/j.jbc.2022.102771

**Published:** 2022-12-05

**Authors:** Alexandra N. Olson, Serena Song, Jonathan D. Dinman

**Affiliations:** Department of Cell Biology and Molecular Genetics, University of Maryland, College Park, Maryland, USA

**Keywords:** eEF2, translation, ribosome, Spinocerebellar ataxia, neurodegeneration, neurocranial development, mutagenesis, frameshifting, fidelity, yeast, LSU, large subunit, PRF, programmed ribosomal frameshifting, SCA, Spinocerebellar ataxias, SRL, sarcin-ricin loop

## Abstract

An emerging body of research is revealing mutations in elongation factor eEF2 that are implicated in both inherited and *de novo* neurodevelopmental disorders. Previous structural analysis has revealed that most pathogenic amino acid substitutions map to the three main points of contact between eEF2 and critical large subunit rRNA elements of the ribosome, specifically to contacts with Helix 69, Helix 95, also known as the sarcin-ricin loop, and Helix 43 of the GTPase-associated center. In order to further investigate these eEF2–ribosome interactions, we identified a series of yeast eEF2 amino acid residues based on their proximity to these functionally important rRNA elements. Based on this analysis, we constructed mutant strains to sample the full range of amino acid sidechain biochemical properties, including acidic, basic, nonpolar, and deletion (alanine) residues. These were characterized with regard to their effects on cell growth, sensitivity to ribosome-targeting antibiotics, and translational fidelity. We also biophysically characterized one mutant from each of the three main points of contact with the ribosome using CD. Collectively, our findings from these studies identified functionally critical contacts between eEF2 and the ribosome. The library of eEF2 mutants generated in this study may serve as an important resource for biophysical studies of eEF2/ribosome interactions going forward.

Protein synthesis (translation) is a core cellular process. At its heart is the ribosome, a universally conserved, two subunit poly-amino acid polymerase that decodes the genetic information encoded in mRNAs into proteins. Once initiated on an mRNA, the ribosome proceeds through a cyclic function called elongation, which is facilitated by two *trans*-acting factors: 1) a ternary complex consisting of elongation factor 1 (EF-Tu in prokaryotes, eEF1A in eukaryotes) complexed with an aminoacyl-tRNA and GTP and 2) a translocase consisting of elongation factor 2·GTP (EF-G·GTP and eEF2·GTP, respectively). The ternary complex delivers new amino acids to the nascent peptide chain on the ribosome, after which the translocase moves the ribosome down the mRNA in the 3′ direction by one codon in a process called translocation.

During translocation, the tRNAs in the P/E and A/P hybrid states fully shift to the E and P sites on both subunits, respectively, and the small subunit moves one codon in the 3′ direction positioning its unoccupied A site to decode the next codon. While inherent ribosome rotations are able to translocate without catalysis, the presence of eEF2·GTP increases the rate by 10^4^-fold (1). It does so by accommodating into the A site and inducing ribosomal conformation changes that accelerate the process. Because of its necessity for efficient translocation, eEF2 may play a role in accurately decoding an mRNA, referred to as translational fidelity. As the ribosomal contacts with the tRNA–mRNA complex are reoriented during translocation, it presents the most vulnerable state for slippage of the ribosome into an aberrant reading frame, that is, frameshifting. eEF2 is thought to not only enhance the speed of translocation but its accuracy as well. Indeed, structural studies have suggested that a unique diphthamide posttranslational modification acts as a “pawl” that prevents the ribosome from slipping out of frame during translocation (2) as well as “unlocking” the codon–anticodon complex from the decoding center, thereby breaking the hold the ribosome has on the tRNA–mRNA complex (3). Additionally, the recent elucidation of a chimeric elongation complex without EF-G demonstrated that in its absence, tRNA and mRNA movement become uncoupled (4) and it has been shown that EF-G also accelerates conformational changes that secure the new ribosomal contacts with the tRNA–mRNA complex after the completion of translocation (5).

Given the centrality of translation, it was long believed that mutations in components of the translational apparatus would be incompatible with successful completion of highly coordinated and complex vertebrate developmental programs. This view was challenged with the discovery that Diamond-Blackfan Anemia is caused by mutations in a ribosomal protein (6). With the advent of improved sequencing technologies, the list of “ribosomopathies” has continued to expand: the current list of mutations responsible for this class of disorders includes genes encoding ribosomal proteins, ribosome biogenesis factors, and posttranscriptional rRNA-modification complexes (7). Ribosomopathies can manifest as a variety of phenotypes, all characterized by developmental defects due to cell hypoproliferation, including anemia and/or craniofacial malformations (8, 9). Confoundingly, this class of patients are also more susceptible to various cancers later in life, indicating that there is a cellular transition from a hypoproliferative state to a hyperproliferative one (9). This switch, known as “Damashek’s Riddle” (10), is thought to be driven by a Darwinian process that selects for secondary mutations that bypass the decreased fitness caused by the original ribosomopathic mutation (9).

Since translation relies on many interacting molecules besides the ribosome, it is possible that mutations to—or biogenesis defects in—other components of the translational apparatus could also result in disease. Accordingly, in tandem with the increased availability of patient genome sequencing, a growing list of genes either directly involved in or adjacent to translation have been implicated in genetic disorders. These include mutations in tRNA processing (6, 7) and tRNA modification proteins (11, 12, 13, 14), aminoacyl-tRNA synthetases (15, 16), and in the translation elongation factors (17, 18, 19, 20, 21, 22, 23). These “translationopathies” tend to share common clinical features including developmental abnormalities and neurological defects and, on a molecular level, loss of translational fidelity (24).

Spinocerebellar ataxias (SCAs) are a largely heterogenous group of genetic diseases characterized by progressive degeneration of the cerebellum, leading to loss of muscle coordination and a variety of other symptoms (25). Each of the 31 different subtypes of SCA result from mutations at distinct genetic loci, indicating that Purkinje neurons, which form the cortex of the cerebellum, are particularly susceptible to genetic defects. These neurons appear to be more sensitive to proteotoxic stress than other cell types, presumably due to their large size, branched morphology, and their susceptibility to being overloaded by misfolded proteins (26). The autosomal dominant disorder Spinocerebellar ataxia 26 (SCA26) is caused by mutation of proline at position 596 to histidine (P596H) in the gene encoding eEF2, which results in increased frequency of translational errors (17). It is hypothesized that eEF2-P596H causes cerebellar degeneration by disrupting translational fidelity, resulting in accumulation of misfolded proteins (*i.e.*, proteotoxic stress) and degeneration of Purkinje neurons.

Additional patients harboring novel eEF2 mutations have been identified (27). In contrast to P596H, these eEF2 mutations including V28M, C372Y, and H769Y were associated with neurodevelopmental delays and cranial abnormalities, indicating that eEF2 mutations have wider-reaching pathologies than SCA26. Structural analysis revealed that almost all of these pathogenic amino acid substitutions map to the three main points of contact between eEF2 and critical rRNA elements of the ribosome. Specifically, the eEF2 mutant P596H mapped to contacts with H69, V28M with the sarcin-ricin loop (SRL, H95), and H769Y with H43 of the GTPase-associated center, all of the large subunit (LSU) of the ribosome (Fig. 1). Each of these ribosomal sites are highly conserved and have been implicated in various functions. The GTPase-associated center is responsible for activating the GTPase activity of translation factors, while the SRL is involved in linking GTP hydrolysis to ribosome conformational changes. H69 is part of the intersubunit bridge, which helps to link the large and small subunits of the ribosome and interacts with several translation factors and tRNA in the A site. They have each been implicated as having functions in translocation, but the relevance of their interactions with eEF2 are not well understood.

**Figure 1:**
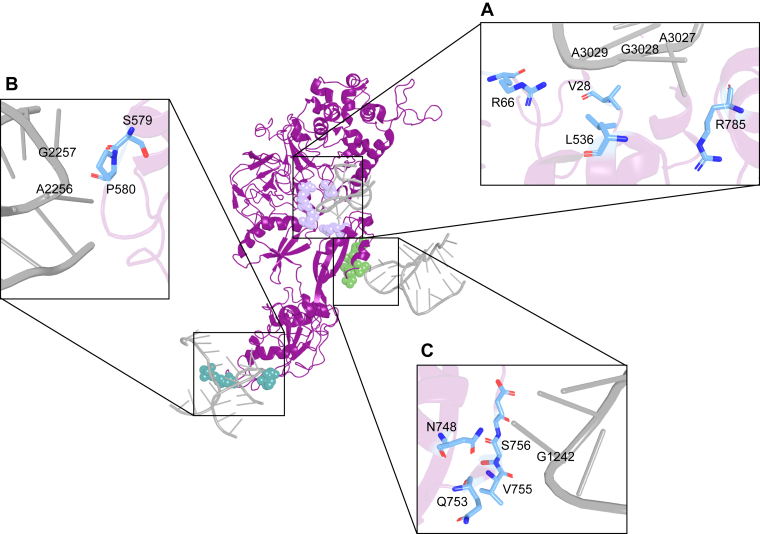
**eEF2-interaction sites with the ribosome.** Structure of *Saccharomyces cerevisiae* bound to the ribosome (modified from PDB 6GQV(43)) eEF2 shown in *purple*, eEF2 residues in *light purple*, *green*, *teal*, and *blue*, and rRNA in *light gray*. *A*, residues proximal to the sarcin-ricin loop. *B*, residues proximal to H69 in the A site of the SSU. *C*, residues proximal to H43 in the GTPase-associated center.

In the current study, a large number of yeast eEF2 mutants were generated based on their proximity to these functionally important rRNA elements in order to further investigate the contributions of these eEF2–ribosome interactions to translational fidelity. The targeted residues were constructed as to test the biochemical contributions of amino acid side chains, including size, charge and polarity on cell growth, viability, sensitivity to translational inhibitors, and translational fidelity. The effects of a subset of mutants on gross eEF2 structure and ribosome-stimulated GTPase activity were also preliminarily investigated.

## Results

### Alterations to eEF2 sites of ribosome interaction are lethal and confer growth defects in yeast models

**Figure 2:**
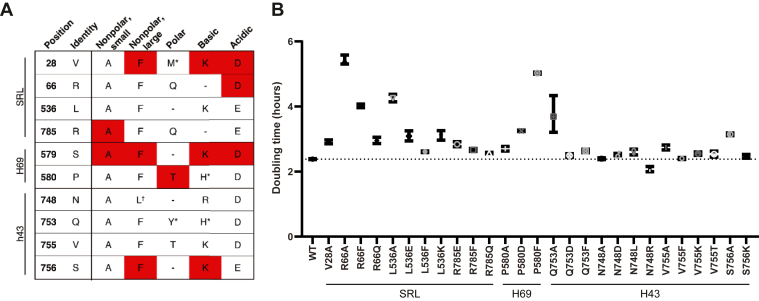
**eEF2-interaction sites are necessary for cell viability and normal growth.***A*, rational design approach to eEF2 mutational analysis. Target residues are listed along with the variants made according to their chemical properties. *Shaded cells* indicate strains found to be inviable. *Dashes* represent mutations which would recapitulate the biochemical properties of the human sidechain and were therefore excluded. ∗Mutants described in previous research. ^+^Nonpolar variant of equivalent size to original human sidechain. *B*, doubling times of viable eEF2 mutant strains. Doubling times were calculated by nonlinear regression of the exponential phase of growth of n = 3 experiments for each strain. Error bars represent 95% C.I. *Dashed line* indicates doubling time of the WT strain (2.38 h).

*EFT2* mutant yeast strains were generated from a parent strain in which the endogenous *EFT1* and *EFT2* genes were disrupted and replaced by a centromeric plasmid encoding either WT or mutant *EFT2* under control of its endogenous promoter. Standard auxotrophic and 5-FOA–driven plasmid shuffle methods were used to select for cells expressing the eEF2 mutants. This method also enabled the identification of lethal mutants of this essential protein (Fig. 2*A*). For each of the three rRNA interaction sites, there was at least one amino acid to which substitutions were particularly deleterious. Three out of the four mutations to V28 in the SRL-interacting site, V28F, V28K, and V28D, substitutions were lethal, suggesting that sidechain length rather than charge properties are most important at this position. A similar trend was observed for residue 66. Conversely, charge appeared to be the critical criterion at residue 785. For the H69 residues, all substitutions of S579 were lethal, while almost all substitutions to P580 were tolerated. Mutations of amino acids that interact with H43 interactions appeared to be less deleterious overall, but two substitutions at S756 were inviable. Other residues were able to tolerate all but one substitution, including R66D and R785A of the SRL-proximal residues and P580T of the H69 group.

Growth rates of the viable yeast strains were first determined to begin characterizing the global effects of these eEF2 mutations (Fig. 2*B*). Most mutations conferred small decreases in growth rates, resulting in a marginally longer doubling times. Several positions proximal to the SRL appeared to increase the doubling times to a greater extent, including R66A, R66F, and L536A. Of the single remaining H69-interacting residue, only P580F exhibited more than a 2-fold increase in doubling time. Of the amino acids that interact with H43, only Q753A significantly affected doubling time.

### Rationally designed eEF2 variants demonstrate sensitivity to decoding center inhibitors

**Figure 3:**
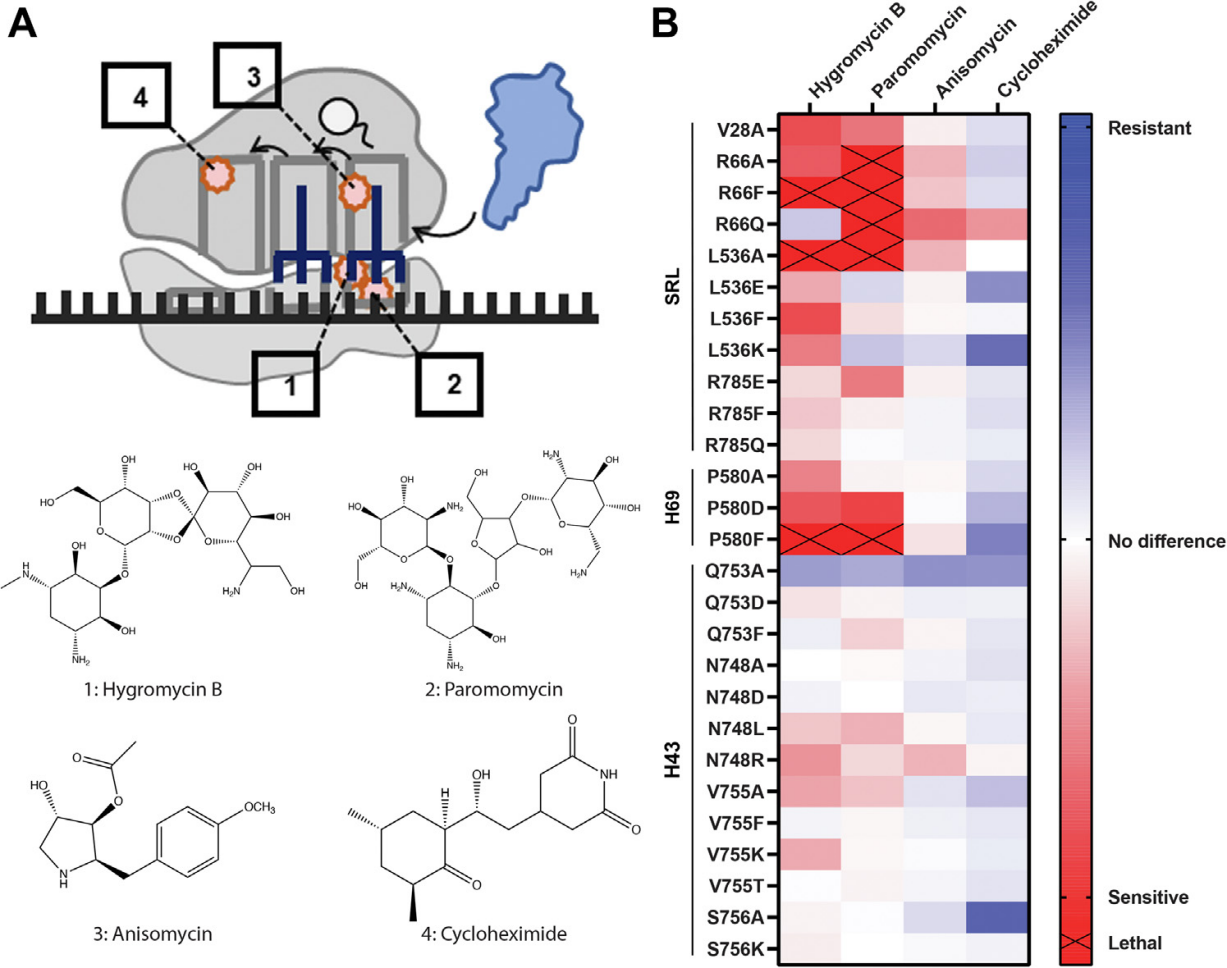
**eEF2 mutations to ribosome-interaction sites confer sensitivity to aminoglycosides.***A*, diagram of antibiotic interactions with the ribosome and their structures. Hygromycin B (1) and paromomycin (2) bind to the A site of the SSU. Anisomycin (3) binds to the A site of the LSU. Cycloheximide (4) binds to the LSU in the E site. Created with Biorender.com. *B*, heat map of eEF2 mutant strain growth in the presence of translational inhibitors. Color saturation indicates strength of the phenotype indicated. Xs indicate strains that were unable to grow at the concentration of inhibitor used. Heat map values calculated by the log_2_ of the ratio of mutant doubling time to WT doubling time in the presence of translational inhibitor (n = 3), minus the log_2_ of the ratio of mutant doubling time to WT doubling time in rich media. Positive values indicate resistance, negative values indicate sensitivity, and 0 indicates no difference in growth due to translational inhibitor. LSU, large subunit.

In order to probe the effect of eEF2 mutations on critical sites of interaction between eEF2 and the ribosome, cellular growth in the presence of sublethal concentrations of ribosome-targeting translational inhibitors were assayed (Fig. 3). Hygromycin B and paromomycin are aminoglycosides that bind to rRNA near the decoding center in the A site of the small ribosomal subunit (SSU) (Fig. 3*A*). Specifically, paromomycin stabilizes the binding of near-cognate tRNAs in the A site, inducing missense incorporation and inhibiting translocation, while hygromycin B also stabilizes A site tRNA as well as interfering with the conformational changes associated with rotation of the ribosome (28, 29). Anisomycin binds the peptidyltransferase center in the A site of the LSU (Fig. 3*A*), where it destabilizes aa-tRNA binding and inhibits peptidyl transfer (27, 28). Cycloheximide binds to the LSU E site (Fig. 3*A*) competing with deacylated tRNA and inhibiting the movement of the P site tRNA into the E site during translocation (30). By measuring the doubling times of the eEF2 variants in the presence of these translation inhibitors, the degree to which the difference between the WT and mutant changes between normal and inhibited growth can be compared, indicating how specific mutations may alter the functional interactions between eEF2 and the ribosome. As shown in Figure 3*B* (and Table S6), inhibitors hygromycin B and paromomycin tended to slow the growth rate of the mutant strains, with some being unable to survive at the concentrations used. This indicates that most of the mutants functionally perturb the interactions between eEF2 and the decoding center. Anisomycin had little to no effects, consistent with the lack of interactions between eEF2 and the peptidyltransferase center A site. Most of the mutants conferred at least some degree of resistance to cycloheximide, consistent with the roles played by movement of deacylated tRNA into the E site prior to translocation. The ribosome interaction sites also tended to confer distinct profiles, with the residues that interact with the SRL and H69 showing the strongest differences. For the SRL proximal site, all R66 mutants were inviable in the presence of paromomycin, and R66F and L536A were inviable with both aminoglycosides. V28A also demonstrated sensitivity to both drugs, while the L536 mutants had moderate, variable responses and some of the strongest resistance to cycloheximide. At the H69-interaction region, P580 residues exhibited the same patterns of growth, with the strength of the phenotype increasing from P580A to D to F, the latter of which was also inviable in the presence of both aminoglycosides. Interestingly, residues near H43 showed little to no growth alterations in the presence of translation inhibitors, with the notable exceptions of Q753A, which conferred resistance to all four antibiotics, and S756A promoting strong resistance to cycloheximide.

### Effects of mutations in eEF2–ribosome interactions on translational fidelity

To determine if changes to these eEF2–ribosome interactions alters eEF2’s ability to promote accurate translocation, frameshifting directed by programmed ribosomal frameshifting (PRF) signals were measured. PRF signals are *cis*-acting elements that induce ribosomal pausing over a sequence that is prone to slippage. The most common PRF signals induce frameshifting of one nucleotide in either the 5′ or 3′ direction, referred to as −1 and +1 frameshifting, respectively. It has been shown that endogenous rates of PRF are controlled by kinetic partitioning between translocation intermediates (31). Therefore, changes to translocation caused by eEF2 mutation can be measured as deviations from the baseline proportion of frameshifting events.

**Figure 4:**
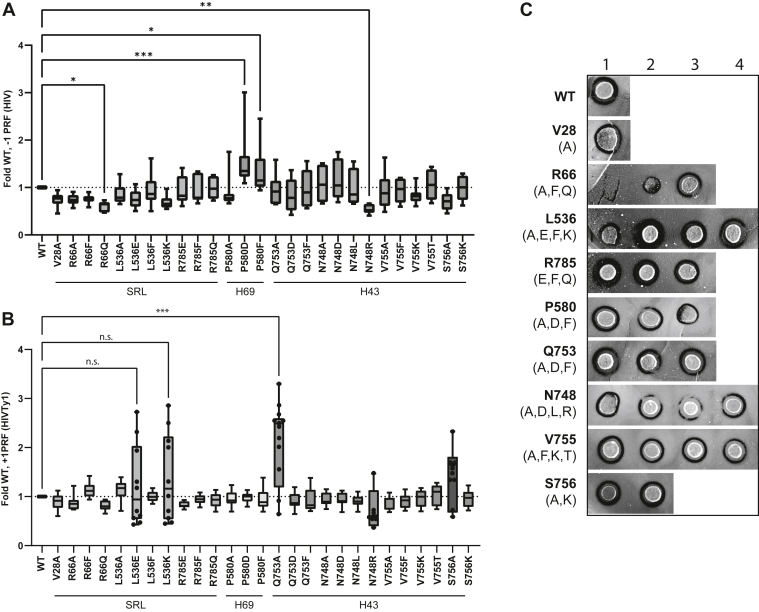
**Translational fidelity defects resulting from eEF2 mutations to ribosome-interaction sites.** Effects of eEF2 mutants on L-A virus-directed −1 PRF (*A*) and Ty*1*-directed +1 PRF by (*B*). Data represented as fold-change values relative to WT. Box plots show the median, interquartile range, and range of data. *p*-values were calculated by Tukey’s multiple comparisons tests between the WT and each variant (n = 8) ns: non-significant, ∗*p* < 0.05, ∗∗*p* < 0.01, ∗∗∗*p* < 0.001. *C*, killer virus maintenance assay of eEF2 mutant strains. Killer activity exhibited by a zone of inhibition (*black rings*) surrounding the spotted yeast strain. Loss of killer activity indicates translational fidelity loss rendering Killer virus replication and therefore toxin production. Representative results from n = 4 shown. Error bars in Fig. S1 denote SD as calculated using student’s *t* test. PRF, programmed ribosomal frameshifting.

The effects of the mutants on programmed translational recoding were measured using dual luciferase assays of HIV-1–mediated −1 PRF and Ty*1*-driven +1 PRF as previously described (27). Briefly, production of the downstream firefly luciferase enzymes is reliant on successful ribosomal frameshifting, while upstream *Renilla* luciferase functions as an internal control for any effects on general translation conferred by the mutants. Therefore, the ratio between the two luciferase activities, as measured by luminescence, is reflective of the fraction of times frameshifting occurs along this message. As shown in Figure 4*A*, the H69 mutants conferred the strongest effects on −1 PRF, with P580D and P580F demonstrating significant increases. Conversely, the R66Q and N748R mutants conferred moderate but significant decreases on −1 PRF. The L536E, L536K, and Q753A strains of the SRL and H43 regions promoted increases in +1 PRF, while the H69 mutations did not affect this translational recoding mechanism (Fig. 4*B*). We note, however, that the data tended to show a bimodal distribution, with clusters of high and low frameshift values. Therefore, only the Q753A strain had a statistically significant increase in frameshifting.

As changes to frameshifting were seen using the translational fidelity reporters, translocation fidelity was further explored using an orthogonal method. Many commercial and research yeast strains are carriers of the L-A totivirus, a dsRNA virus that supports the M1 satellite virus, which encodes for the “killer toxin” which is lethal to noncarrier strains (32). The L-A virus genome contains a −1 PRF signal at the *Gag/gag-pol* juncture necessary for viral replication (33). Maintenance of the Killer phenotype is highly dependent on −1 PRF efficiency (34); therefore, defects in frameshifting will materialize as reduction or loss of viral persistence and killer toxin production. The parental strain used in these studies (yJD995) has the Killer^+^ (K^+^) phenotype, thus enabling us to determine the effects of the eEF2 mutants on viral maintenance. To monitor this, yeast strains are grown on a lawn of the diploid 5 × 47 K^−^ indicator strain that is sensitive to the toxin, and the size of the zone of growth inhibition around the strain being tested is indicative of cellular M1 viral load. The R66A mutant was completely unable to maintain the Killer virus, and the R66F, L536A, P580D, and P580F showed partial decreases in killer activity, suggesting partial defects on virus maintenance (Fig. 4*C*). Quantitative analysis of the zones of growth inhibition are shown in Fig. S1. Given the relatively small effects of the mutants on −1 PRF in general, it is likely that the effects of these mutants on killer virus maintenance correlated better with decreased fitness as reflected by increased cell doubling times (compare with Fig. 2*B*).

### Effects of selected mutants on eEF2 gross structure and GTPase activity

Based on the phenotypes displayed in the growth and translational fidelity assays, one mutation was selected from each cluster for deeper biophysical and biochemical characterization. R66A, P580H, and Q753A were chosen from the SRL, H69, and H43 regions, respectively. P580H, which was previously described in greater detail (17), is the original eEF2 mutation found to be causative for SCA26 and has previously demonstrated phenotypes similar to those expressed by P580D and P580F. Therefore, due to its known biological significance and characterization by several groups, P580H was chosen to represent the H69-interacting region.

**Figure 5:**
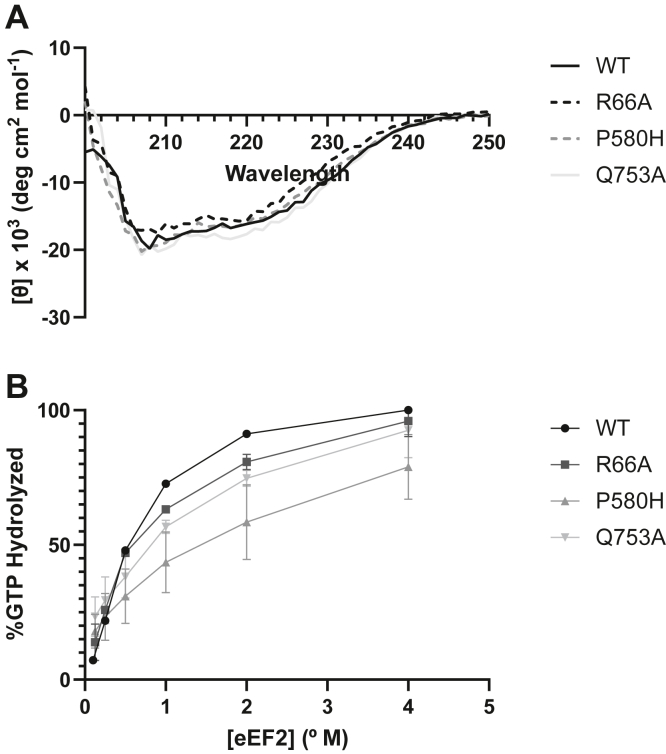
***In vitro* characterization of selected eEF2 mutants.***A*, circular dichroism spectra of WT and mutant eEF2. *B*, *in vitro* GTPase activity of WT and mutant eEF2. Luminescence data was normalized to concurrently measure positive and negative controls. WT n = 1, all mutants n = 2.

CD spectra were obtained for purified WT and three mutant eEF2s to determine whether the mutations imparted biophysical effects on eEF2 folding (Fig. 5*A*). The mutants all exhibited similar CD spectra to the WT protein, indicating that none conferred gross defects on eEF2 structure. This is consistent with their ability to support cell viability as the sole form of the protein. However, an increase in lower molecular weight contaminants was noticed during purification of R66A and P580H. These appeared in middle stages of purification and were subsequently removed during the final preparation step (Fig. S2*B*). This could indicate higher amounts of eEF2 degradation, potentially as a result of folding defects. However, since these lower MW species were removed during purification, they were not represented in the CD spectra.

To examine the functional effects of the eEF2 mutations, the ability of the purified eEF2 samples to hydrolyze GTP was measured in the presence of 80S ribosomes (Fig. 5*B*). R66A and Q753A showed moderate reductions in GTPase activity, while P580H showed a marked decrease in GTP hydrolysis. These experiments were only able to be carried out once for WT and twice for mutant eEF2 due to material constraints. Therefore, these data should be viewed as preliminary and are included here to highlight an interesting future direction for investigation into eEF2 mutations. Even with their limitations, these experiments indicate that in the absence of gross folding differences, mutations of eEF2 that map to sites of interaction with the ribosome can directly impact the enzymatic activity of this protein, suggesting that they may impair translocation rates.

## Discussion

The cost of DNA sequencing has decreased to a level where it is becoming an effective tool for diagnosis of rare genetic disorders. Accordingly, missense mutations of eEF2 are emerging as the underlying causes of an increasing number of neurodevelopmental pathologies (17, 23, 27). Importantly, many of these have all mapped to the three sites where eEF2 physically interacts with functionally important rRNA-based structural features of the ribosome: The SRL (H98), H69, and the H43 of the GTPase-associated center. The current study employed deep mutagenesis of eEF2 amino acid residues involved in these biophysical interactions to probe the importance of eEF2–ribosome interactions on cellular fitness and translational fidelity. One general observation is that the antibiotic analysis presented in Figure 3 is consistent with prior observations of allosteric interactions between the ribosomal A- and E-sites (35), as sensitivity to A-site inhibitors correlated with resistance to E-site inhibitors. Additionally, consistent with a prior study of yeast eEF2 mutants (36), most of the new alleles also did not affect Killer virus maintenance.

The SRL is a highly conserved region of the 28S rRNA that, along with the GTPase-associated center, coordinates GTPase activity by translation factors with various ribosomal functions, including delivery of cognate aa-tRNAs by the eEF1A–tRNA–GTP ternary complex, translocation by eEF2, termination by the eRF1–eRF3 complex, and interactions with a growing list of factors involved in the ribosome quality control mechanism (37). The SRL has previously been implicated as a key site for EF-G and eEF2 function; though it has been demonstrated that it is not necessary for GTP hydrolysis, it is thought to serve as an anchoring point for EF-G to the ribosome during translocation (37). This is supported by data obtained in eukaryotic systems, with structural studies showing that dissociation of eEF2 from the ribosome is mediated by contacts with the SRL (38). Here, we have demonstrated that eEF2 residues V28, R66, and L536 are important sites of interaction with the SRL. In particular, alterations to V28 were almost always lethal, with the exceptions of substitution with alanine, an amino acid that is similarly small and nonpolar, and the previously investigated methionine substitution (27) that, while having different charge properties, is similar in size to valine. Viable substitutions to the SRL-interacting region promoted sensitivity to aminoglycosides and moderate translational fidelity defects, and *in vitro* characterization of R66A revealed a slight reduction of GTPase activity. Together, these findings identify additional critical contacts between eEF2 and the SRL that are important for translocation and reading frame maintenance.

The GTPase-associated center is comprised of Helices 43 and 44 of the 28S rRNA. eEF2 residues proximal to H43 did not generally promote strong phenotypes, with the exceptions of Q753A and S756A. Q753 is also a position that was previously shown to be linked to neurodevelopmental disorders (27). The Q753A mutation promoted a strong growth defect and increased +1 frameshifting. Most notably, this was the only mutant that did not show the reciprocal relationship between A- and E-site inhibitors. This suggests that the allosteric coordination between these two tRNA-binding sites may also be mediated by the interaction of the ribosome with elongation factors and not simply an intrinsic feature of the LSU. S756A also displayed mild growth defects and conferred strong resistance to cycloheximide but did not alter translational fidelity. In this way, it appears that these two variants have similar phenotypes, with the Q753A defects being more pronounced, indicating that they share a common mechanistic effect. Q753A also showed a similar decrease in GTPase activity similar to the R66A mutant, which is expected as interaction with the GTPase-associated center is important for GTPase activation.

The final region of eEF2 interaction investigated was with H69 of the LSU. This helix is part of the B2a intersubunit bridge and has been implicated in the “unlocking” mechanism of the decoding center from the tRNA–mRNA complex. Particularly, rearrangement of A2256 (Fig. 1*B*) in H69 has been shown to be part of the conformational change that destabilizes the contacts between h44 of the decoding center and the codon–anticodon interaction (39). Mutations of the two residues close to H69 in this work further underscore the importance of this function to reading frame maintenance. The mutations attempted at position S579 in this work were all lethal, indicating essential nature of this amino acid residue. P580 is the location of the original SCA26 allele and the additional variants generated in this work recapitulated its effects from previous research to varying extents. P580H promoted the strongest defect in the GTPase activity of eEF2, possibly due to decreased binding affinity with the A site or defects in the allosteric network to trigger GTP hydrolysis. The necessity of S579 and translational fidelity loss seen with several P580 mutations indicate these two residues as an important interaction site with A2256 and the subsequent unlocking mechanism that coordinates reading frame maintenance.

Overall, several original residues recapitulate the phenotypes of yeast strains modeled after alleles implicated in neurological translationopathies (27), supporting the hypothesis that alterations of these eEF2-ribosome contacts are part of the disease mechanism. This, combined with the data on translational inhibitor sensitivity, translational fidelity, and ribosome-stimulated GTPase activities further refine our understanding of the importance of eEF2-ribosome contacts.

## Experimental procedures

### Yeast strains and media

For the construction of *EFT2*-6xHis mutational analysis strains, site-directed mutagenesis of pJD2490 (YCp*EFT2-6xHIS*-*LEU2*) (27) using synthetic oligonucleotides (Table S1) was performed using Q5 high-fidelity DNA polymerase (NEB) per the manufacturer’s instructions with the following adjustments. Four millimolar MgCl_2_ was added to the reaction mixture and touchdown cycling was used to eliminate the need for different cycling parameters for each oligo pair, where the annealing temperature started at 70 °C and decreased 1 °C each cycle for 15 cycles, then remained at 54 °C for the remaining 20 cycles. Template DNA was then digested in concert with phosphorylation and circularization of PCR product. One microliter of PCR reaction mixture was incubated with 5U of T4 DNA ligase (NEB), 200U of T4 polynucleotide kinase (NEB), and 10U of DpnI (NEB) in T4 DNA ligase buffer (NEB) at a final volume of 10 μl. The reaction mixture was incubated at room temperature for 2 h, then at 30 °C for 30 min. The final reaction mixture was transformed into NEB 5-alpha competent *Escherichia coli* (high efficiency) per manufacturer’s instructions, and transformed cells were plated onto LBCb_50_ plates. Mutagenesis was confirmed by diagnostic digest of the transformed plasmid by Pst I-FD (Thermo Fisher Scientific) and Sanger sequencing by Genewiz using the oligonucleotides listed in Table S2.

*EFT2* mutant plasmids were transformed into yJD995 (*MATa ade2 ura3 his3 leu2 trp1 eft::HIS3 eft2::TRP1* +YCp*EFT1*-*URA3*) as previously described (27) except confirmation of successful transformation was determined by colony PCR using the oligonucleotides listed in Table S3. Briefly, 50 μl of transformed yeast culture was pelleted and resuspended in water, which was then incubated at 99 °C for 5 min. “Heat popped” yeast were then used as template DNA for PCR as 1/10 of the total reaction volume using DreamTaq MasterMix (Thermo Fisher Scientific) and appropriate primers. PCR product was purified with DNA Clean & Concentrator kit (Zymo) and sequenced by Genewiz using Sanger sequencing to confirm incorporation of mutant *EFT2*. A list of all plasmids used in this study is shown in Table S4.

### Yeast strain growth analysis

Yeast strains transformed with plasmids (Table S5) were grown to logarithmic phase then diluted to an A_600_ = 0.05 in either YPAD only or YPAD with translation inhibitor. Diluted cultures were split into three wells of a clear 96-well plate which was then sealed with a Breathe Easy sealing membrane (Electron Microscopy Sciences). Yeast were grown in a Synergy HTX plate reader (BioTek) at 30 °C for 48 h. The A_600_ of each well was taken every 15 min, with shaking for 10 min preceding each measurement. Nonlinear regression was performed with GraphPad Prism to fit the exponential phase to an exponential growth function. Doubling times were calculated by dividing ln(2) by the rate parameter found through nonlinear regression, and SDs were calculated using a student’s *t* test. Concentrations of translation inhibitors were determined by an approximate 50% reduction in growth rate of WT *EFT2* yeast. These concentrations were as follows: 15 μg/ml anisomycin, 5 mg/ml paromomycin, 20 μg/ml hygromycin B, and 50 ng/ml cycloheximide.

### Translational fidelity assays

The killer maintenance assay was performed by growing *EFT2* strains to mid log phase overnight in YPAD, then diluting each culture to an A_600_ of 1 in YPAD with 30% glycerol. 5 × 47 (*MATa/MATα his1/+ trp1/+ ura3/+ K*^*−*^ *R*^*−*^) (40) was used as the killer sensitive strain and was grown and diluted the same, except without the presence of glycerol. 10^7^ cells of 5 × 47 were spread onto 4.7 MB plates and allowed to fully dry. 6 × 10^4^ cells of *EFT2* strains were spotted onto lawn plates and were incubated at room temperature (∼20 °C) for 5 days. Dual-luciferase assays were performed as previously described (27). Statistical analyses were performed as previously described (41), data were normalized to fold-WT, and *p*-values were calculated by Tukey’s multiple comparisons tests between the WT and each variant.

### Purification of histidine-tagged eEF2

Yeast cultures were grown in YPAD to an A of 1.5. Cells were harvested by centrifugation and washed twice with equilibration buffer (20 mM sodium phosphate pH = 7.4, 500 mM NaCl, 20 mM imidazole). Cells were lysed by addition of 2.5 to 3 ml of lysis buffer (Yeast Protein Extraction Reagent (Thermo Fisher Scientific), 1× Halt protease inhibitor (Thermo Fisher Scientific), 1 mM DTT) per gram of cells (wet weight) and mixed gently at room temperature for 20 min. Lysate was clarified by centrifugation at 4000*g* for 25 min at 4 °C and then combined with an equal amount of 2× equilibration buffer (40 mM sodium phosphate pH = 7.4, 1 M NaCl, 40 mM imidazole). Lysate was filtered through a 0.22 μm syringe filter then purified on a 1 ml HisTrap HP column (Cytiva) using an AKTA FPLC instrument. The column was washed with 10 volumes of equilibration buffer and then 5 volumes of 10% elution buffer. Protein was eluted with elution buffer (20 mM sodium phosphate pH = 7.4, 500 mM NaCl, 250 mM imidazole) and fractions containing eEF2-6xHis were concentrated with an Amicon Ultra-15 50K centrifugal filter. The concentrated protein was then loaded onto a HiPrep 16/60 S-200 HR column (Cytiva) and isocratically eluted with 20 mM Tris–HCl pH = 7.5, 150 mM NaCl, 0.1 mM EDTA. Fractions containing eEF2-6xHis were again concentrated by centrifugal filtration and stored in HiPrep buffer with 10% glycerol at −80 °C. Purification was evaluated by SDS-PAGE (Fig. S1) and concentration was measured using Coomassie Plus Bradford assay (Thermo Fisher Scientific).

### CD spectroscopy

Purified eEF2 was buffer exchanged into 20 mM Tris–HCl pH = 7.5, 1 mM EDTA and diluted to a final concentration of 0.15 mg/ml. CD spectra were taken in a 0.1 cm quartz cuvette with a JASCO J810 spectro-polarimeter at room temperature from 250 to 200 nm with a scanning speed of 20 nm/min. CD of a blank run containing only buffer was subtracted from each data set, and molar ellipticity was calculated using the exact concentration as measured by Bradford assay.

### GTPase activity measurements

GTPase activity of purified eEF2 was carried out using the GTPase-Glo Assay (Promega) as described (42, 43). A 10 μl reaction containing 0.1 μM 80S ribosomes, 0.5 μM GTP, 1 mM DTT, 0.5 mg/ml polyU, and varying amounts of eEF2 (4–0.125 μM) in GAP/GTPase buffer were incubated at 30 °C for 90 min and remaining [GTP] was converted to luminescence as per the manufacturer’s instructions. Luminescence was measured in a white 384-well plate (Corning) with the Promega GloMax Multi+ Detection system. Relative luminescence units values were normalized to each data set and GTPase activity was calculated as described (43).

## Data availability

All data described here are contained within the article as either a main figure or a supporting figure. Primary data will be made available upon request. Address all inquiries to Jonathan Dinman (dinman@umd.edu).

## Supporting information

This article contains supporting information.

## Conflict of interest

The authors declare that they have no conflicts of interest with the contents of this article.
