# Supplementary material for: Deep mutational analysis of elongation factor eEF2 residues implicated in human disease to identify functionally important contacts with the ribosome
Source: J Biol Chem. 2022 Dec 5;299(1):102771. doi: 10.1016/j.jbc.2022.102771 (PMC9830224; doi:10.1016/j.jbc.2022.102771)
Supplement: Supplemental Tables S1–S6 and Figures S1–S2 [file mmc1.docx]

**Supplementary Information**

Supplementary Table 1: List of oligonucleotides used for site-directed mutagenesis

| Name | Sequence |
| --- | --- |
| V28M-fwd | GTCCGTTATTGCTCACatgGATCATGGTAAGTCCAC |
| V28M-rev | GTGGACTTACCATGATCcatGTGAGCAATAACGGAC |
| C372Y-fwd | CTGTATTGCTATCAAGAACtatGATCCAAAGGCTGATTTGATG |
| C372Y-rev | CATCAAATCAGCCTTTGGATCATAGTTCTTGATAGCAATACAG |
| P580H-fwd | CTCAAACTGCTTTGTCCAAGTCTcatAACAAGCATAACAGAATC |
| P580H-rev | GATTCTGTTATGCTTGTTATGAGACTTGGACAAAGCAGTTTGAG |
| Q753Y-fwd | CGTCTTAAACAAGAAGAGAGGTtatGTCGTTTCTGAAGAACAAAG |
| Q753Y-rev | CTTTGTTCTTCAGAAACGACATAACCTCTCTTCTTGTTTAAGACG |
| Q753H-fwd | CGTCTTAAACAAGAAGAGAGGTcacGTCGTTTCTGAAGAACAAAG |
| Q753H-rev | CTTTGTTCTTCAGAAACGACGTGACCTCTCTTCTTGTTTAAGACG |
| H699N-fwd | catgccgatgctatcaacagaggtggtggtc |
| H699N-rev | gaccaccacctctgttgatagcatcggcatg |
| V28A_fwd | TATTGCTCACgccGATCATGGTAAG |
| V28F_fwd | TATTGCTCACttcGATCATGGTAAG |
| V28D_fwd | TATTGCTCACgacGATCATGGTAAG |
| V28AFD_rev | ACGGACATGTTACGCACA |
| V28K_fwd | TATTGCTCACaagGATCATGGTAAGTCCACTTTG |
| V28K_rev | ACGGACATGTTACGCAC |
| R66A_fwd-2 | TAGTGATACCtgcTTCTTGTTCATCC |
| R66E_fwd-2 | TAGTGATACCttcTTCTTGTTCATCC |
| R66F_fwd-2 | TAGTGATACCaaaTTCTTGTTCATCCTTTC |
| R66Q_fwd-2 | TAGTGATACCttgTTCTTGTTCATCC |
| R66AEFQ_rev-2 | TCAAGTCTACCGCTATTTC |
| R785A_fwd | TGGTGAATTGgcaCAAGCTACTG |
| R785E_fwd | TGGTGAATTGgaaCAAGCTACTG |
| R785Q_fwd | TGGTGAATTGcaaCAAGCTACTG |
| R785F_fwd | TGGTGAATTGtttCAAGCTACTGGTG |
| R785AEQF_rev | GTGAAACCGAAAGATTCG |
| L536A_fwd | TACCGGTGAAgctCATTTGGAAATTTGTTTG |
| L536F_fwd | TACCGGTGAAtttCATTTGGAAATTTG |
| L536K_fwd | TACCGGTGAAaagCATTTGGAAATTTG |
| L536AFK_rev | CCAGCAACGATATGTTCAC |
| L536E_fwd-2 | TTTCCAAATGTTCTTCACCGGTACCAG |
| L536E_rev-2 | TTTGTTTGCAAGATTTGG |
| S579A_fwd | TTTGTCCAAGgctCCAAACAAGC |
| S579A_rev | GCAGTTTGAGAAGATTCACTTTC |
| S579D_fwd | TTTGTCCAAGgatCCAAACAAGC |
| S579K_fwd | TTTGTCCAAGaaaCCAAACAAGC |
| S579V_fwd | TTTGTCCAAGgttCCAAACAAGCATAAC |
| S579C_fwd | TTTGTCCAAGtgtCCAAACAAGC |
| S579DKVC_rev | GCAGTTTGAGAAGATTCAC |
| P580A_fwd | GTCCAAGTCTgcaAACAAGCATAAC |
| P580F_fwd | GTCCAAGTCTtttAACAAGCATAAC |
| P580D_fwd | GTCCAAGTCTgatAACAAGCATAAC |
| P580T_fwd | GTCCAAGTCTacaAACAAGCATAAC |
| P580AT_rev | AAAGCAGTTTGAGAAGATTCAC |
| P580FD_rev | AAAGCAGTTTGAGAAGATTC |
| Q753A_fwd | GAAGAGAGGTgctGTCGTTTCTGAAG |
| Q753F_fwd | GAAGAGAGGTtttGTCGTTTCTGAAG |
| Q753D_fwd | GAAGAGAGGTgatGTCGTTTCTG |
| Q753AFD_rev | TTGTTTAAGACGGAGTAG |
| N748A_fwd | CTCCGTCTTAgctAAGAAGAGAGGTCAAGTCG |
| N748D_fwd | CTCCGTCTTAgacAAGAAGAGAGG |
| N748L_fwd | CTCCGTCTTActaAAGAAGAGAGGTCAAGTCGTTTC |
| N748R_fwd | CTCCGTCTTAagaAAGAAGAGAGGTCAAGTC |
| N748ADLR_rev | TAGATACCACCGACGGCT |
| V755A_fwd | AGGTCAAGTCgctTCTGAAGAAC |
| V755A_rev | CTCTTCTTGTTTAAGACGGAG |
| V755F_fwd | AGGTCAAGTCtttTCTGAAGAAC |
| V755T_fwd | AGGTCAAGTCactTCTGAAGAACAAAG |
| V755K_fwd | AGGTCAAGTCaagTCTGAAGAACAAAGAC |
| V755FTK_rev | CTCTTCTTGTTTAAGACGG |
| S756E_fwd | TCAAGTCGTTgagGAAGAACAAAGAC |
| S756A_fwd | TCAAGTCGTTgctGAAGAACAAAG |
| S756F_fwd | TCAAGTCGTTtttGAAGAACAAAG |
| S756K_fwd | TCAAGTCGTTaaaGAAGAACAAAGACC |
| S756EAFK_rev | CCTCTCTTCTTGTTTAAGAC |

Supplementary Table 2: List of oligonucleotides used for sequencing

| Name | Sequence |
| --- | --- |
| Sc_EFT1_F1 | CACTGTTGACCAAATGCGTTC |
| Sc_EFT1_F2 | GAGCTTTGTTGGAATTGCAAG |
| Sc_EFT1_F3 | GAGAAAGTTCTTGCCAGCTGC |
| Sc_EFT1_F4 | CTCTCCAGTTGTGCAAGTCGC |
| Sc_EFT1_F5 | GCTTTCCAATGGGCTACCAAG |
| Sc_EFT1_F6 | TCTGACCCATTGGACCCAACC |
| Sc_EFT1_R1 | TTGTGGGAAAGCTTGACCACC |
| Sc_EFT1_R2 | GCCATGATTCTAGCTCTGGCC |
| Sc_EFT1_R3 | GTGAGCAGTTTCACTGGTGGTC |
| Sc_EFT1_R4 | TCCAAGTCCTTTTCGTCACCC |
| Sc_EFT1_R5 | TTCGGTTTGGACACAGACACC |
| Sc_EFT1_R6 | GCAATAACGGACATGTTACGC |

Supplementary Table 3: List of oligonucleotides used for colony PCR

| Mutations | Name | Sequence |
| --- | --- | --- |
| V28, R66 | EFT2-A fwd | CTGAAGATTGGGTCCAAGATGA |
|  | EFT2-A rev | CTTTCAGCACTGAAGAGTCCA |
| L536, S579, P580, N748, Q753, V755, S756 | EFT2-C fwd | CCAACAAGAAACCAGCGTAAG |
|  | EFT2-C rev | CAAACTACGTTCCAGGTAAGA |
| R785 | EFT2-D fwd | GTCCAATGGGTCAGAACCTAAA |
|  | EFT2-D rev | CCAAGTCTCCAAACAAGCATAAC |

Supplementary Table 4: List of plasmids used in this study

| Plasmid | Description |
| --- | --- |
| pJD375 | pYDL 0-frame control |
| pJD377 | pYDL +1 PRF (Ty1) |
| pJD378 | pYDL -1 PRF (HIV) |
| pJD433 | pYDL TCR (UGA) |
| pJD2257 | pSGDmod 0-frame control |
| pJD2337 | pSGDmod PEG10 (-1 PRF) |
| pJD2349 | pSGDmod OAZ1 (-1 PRF) |
| pJD2443 | pSGDmod UAA |
| pJD2444 | pSGDmod UGA |
| pJD2445 | pSGDmod UAG |
| pJD2490 | pyJD1334 (S.c.*EFT2*-6xHis, see yJD1334) |
| pJD2419 | S.c.*EFT2* in pyJD1334::V28M |
| pJD2422 | S.c.*EFT2* in pyJD1334∷C372Y |
| pJD2423 | S.c.*EFT2* in pyJD1334∷P580H |
| pJD2424 | S.c.*EFT2* in pyJD1334∷Q753Y |
| pJD2425 | S.c.*EFT2* in pyJD1334∷Q753H |
| pJD2497 | S.c.*EFT2* in pyJD1334∷H699N |
| pJD2533 | S.c.*EFT2* in pyJD1334::V28A |
| pJD2534 | S.c.*EFT2* in pyJD1334::V28D |
| pJD2535 | S.c.*EFT2* in pyJD1334::V28F |
| pJD2536 | S.c.*EFT2* in pyJD1334::V28K |
| pJD2537 | S.c.*EFT2* in pyJD1334::R66A |
| pJD2538 | S.c.*EFT2* in pyJD1334::R66E |
| pJD2539 | S.c.*EFT2* in pyJD1334::R66F |
| pJD2540 | S.c.*EFT2* in pyJD1334::R66Q |
| pJD2541 | S.c.*EFT2* in pyJD1334::R785A |
| pJD2542 | S.c.*EFT2* in pyJD1334::R785E |
| pJD2543 | S.c.*EFT2* in pyJD1334::R785F |
| pJD2544 | S.c.*EFT2* in pyJD1334::R785Q |
| pJD2545 | S.c.*EFT2* in pyJD1334::L536A |
| pJD2546 | S.c.*EFT2* in pyJD1334::L536E |
| pJD2547 | S.c.*EFT2* in pyJD1334::L536F |
| pJD2548 | S.c.*EFT2* in pyJD1334::L536K |
| pJD2549 | S.c.*EFT2* in pyJD1334::S579A |
| pJD2550 | S.c.*EFT2* in pyJD1334::S579C |
| pJD2551 | S.c.*EFT2* in pyJD1334::S579D |
| pJD2552 | S.c.*EFT2* in pyJD1334::S579K |
| pJD2553 | S.c.*EFT2* in pyJD1334::S579V |
| pJD2554 | S.c.*EFT2* in pyJD1334::P580A |
| pJD2555 | S.c.*EFT2* in pyJD1334::P580D |
| pJD2556 | S.c.*EFT2* in pyJD1334::P580F |
| pJD2557 | S.c.*EFT2* in pyJD1334::P580T |
| pJD2561 | S.c.*EFT2* in pyJD1334::Q753A |
| pJD2562 | S.c.*EFT2* in pyJD1334::Q753D |
| pJD2563 | S.c.*EFT2* in pyJD1334::Q753F |
| pJD2564 | S.c.*EFT2* in pyJD1334::N748A |
| pJD2565 | S.c.*EFT2* in pyJD1334::N748D |
| pJD2566 | S.c.*EFT2* in pyJD1334::N748L |
| pJD2567 | S.c.*EFT2* in pyJD1334::N748R |
| pJD2568 | S.c.*EFT2* in pyJD1334::V755A |
| pJD2570 | S.c.*EFT2* in pyJD1334::V755F |
| pJD2571 | S.c.*EFT2* in pyJD1334::V755K |
| pJD2572 | S.c.*EFT2* in pyJD1334::V755T |
| pJD2573 | S.c.*EFT2* in pyJD1334::S756A |
| pJD2574 | S.c.*EFT2* in pyJD1334::S756E |
| pJD2575 | S.c.*EFT2* in pyJD1334::S756F |
| pJD2576 | S.c.*EFT2* in pyJD1334::S756K |

Supplementary Table 5: List of yeast strains used in this study

| Strain | Genotype |
| --- | --- |
| yJD995 | (YEFD12h^215^) MATa *ade2 ura3 his3 leu2 trp1 eft1::HIS3 eft2::TRP1* +YCp*EFT1*-*URA3* (*EFT2* parent strain, generously provided by Dr. Terri Kinzy) |
| yJD1334 | (TKy675^28^) MATα *ade2 leu2 ura3 his3 trp1 eft::HIS3 eft2::TRP1* pEFT2-*LEU2*-CEN (*EFT2*-6xHis plasmid isolated and used for mutagenesis (pJD2490), strain generously provided by Dr. Terri Kinzy) |
| yJD1923 | yJD995 + pJD2490_*EFT2*-WT |
| yJD1924 | yJD995 + pJD2419_*EFT2*-V28M |
| yJD1927 | yJD995 + pJD2422_*EFT2*-C372Y |
| yJD1928 | yJD995 + pJD2423_*EFT2*-P580H |
| yJD1929 | yJD995 + pJD2423_*EFT2*-Q753Y |
| yJD1930 | yJD995 + pJD2425_*EFT2*-Q753H |
| yJD1958 | yJD995 + pJD2497_*EFT2*-H699N |
| yJD2026 | yJD995 + pJD2533_*EFT2*-V28A |
| yJD2027 | yJD995 + pJD2537_*EFT2*-R66A |
| yJD2028 | yJD995 + pJD2539_*EFT2*-R66F |
| yJD2029 | yJD995 + pJD2540_*EFT2*-R66Q |
| yJD2030 | yJD995 + pJD2542_*EFT2*-R785E |
| yJD2031 | yJD995 + pJD2543_*EFT2*-R785F |
| yJD2032 | yJD995 + pJD2544_*EFT2*-R785Q |
| yJD2033 | yJD995 + pJD2545_*EFT2*-L536A |
| yJD2034 | yJD995 + pJD2546_*EFT2*-L536E |
| yJD2035 | yJD995 + pJD2547_*EFT2*-L536F |
| yJD2036 | yJD995 + pJD2548_*EFT2*-L536K |
| yJD2037 | yJD995 + pJD2554_*EFT2*-P580A |
| yJD2038 | yJD995 + pJD2555_*EFT2*-P580D |
| yJD2039 | yJD995 + pJD2556_*EFT2*-P580F |
| yJD2040 | yJD995 + pJD2561_*EFT2*-Q753A |
| yJD2041 | yJD995 + pJD2562_*EFT2*-Q753D |
| yJD2042 | yJD995 + pJD2563_*EFT2*-Q753F |
| yJD2043 | yJD995 + pJD2564_*EFT2*-N748A |
| yJD2044 | yJD995 + pJD2565_*EFT2*-N748D |
| yJD2045 | yJD995 + pJD2566_*EFT2*-N748L |
| yJD2046 | yJD995 + pJD2567_*EFT2*-N748R |
| yJD2047 | yJD995 + pJD2568_*EFT2*-V755A |
| yJD2048 | yJD995 + pJD2570_*EFT2*-V755F |
| yJD2049 | yJD995 + pJD2571_*EFT2*-V755K |
| yJD2050 | yJD995 + pJD2572_*EFT2*-V755T |
| yJD2051 | yJD995 + pJD2573_*EFT2*-S756A |
| yJD2052 | yJD995 + pJD2576_*EFT2*-S756K |

Supplementary Table 6: Doubling times of *EFT2* mutant yeast strains under standard conditions and in the presence of translational inhibitors.


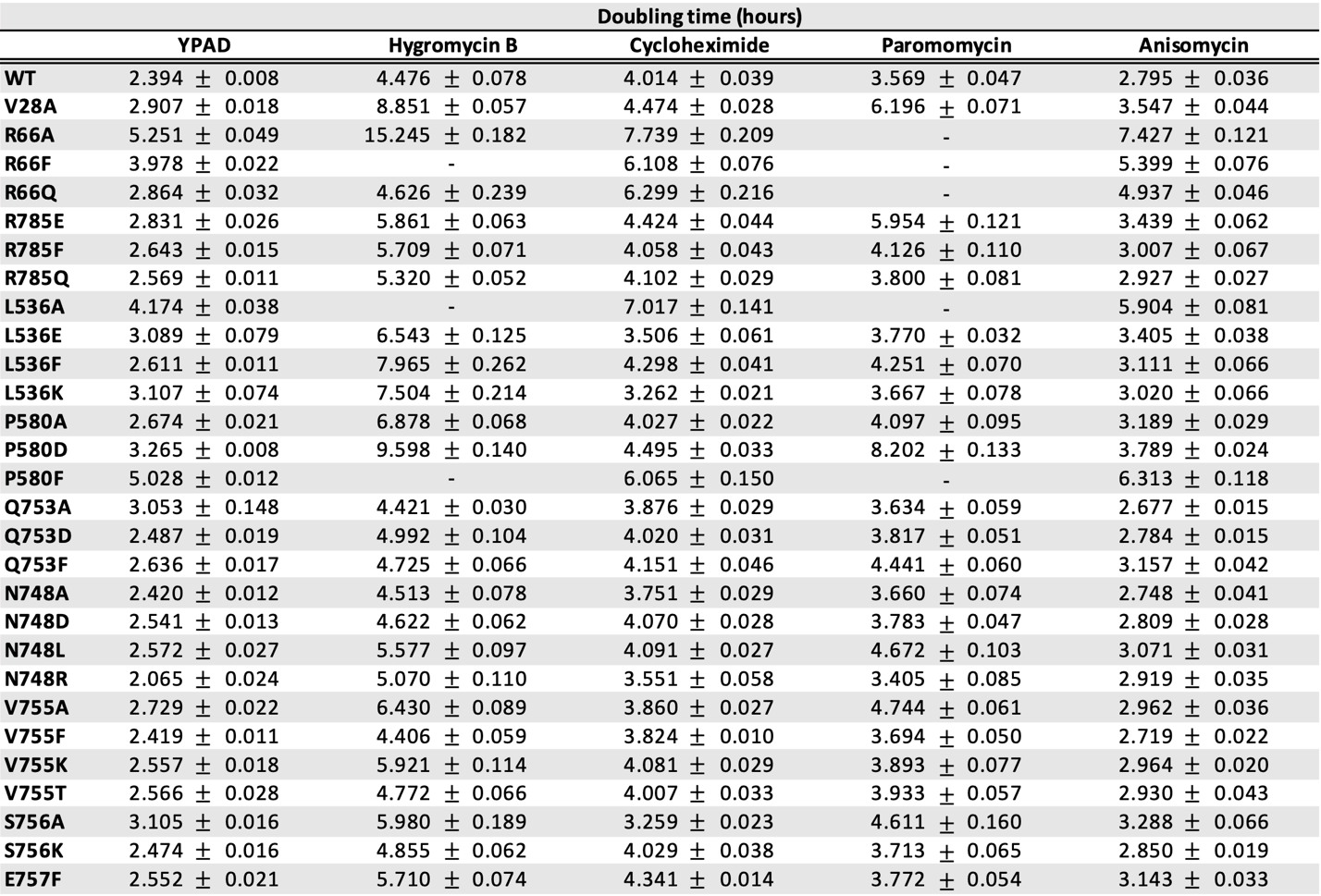


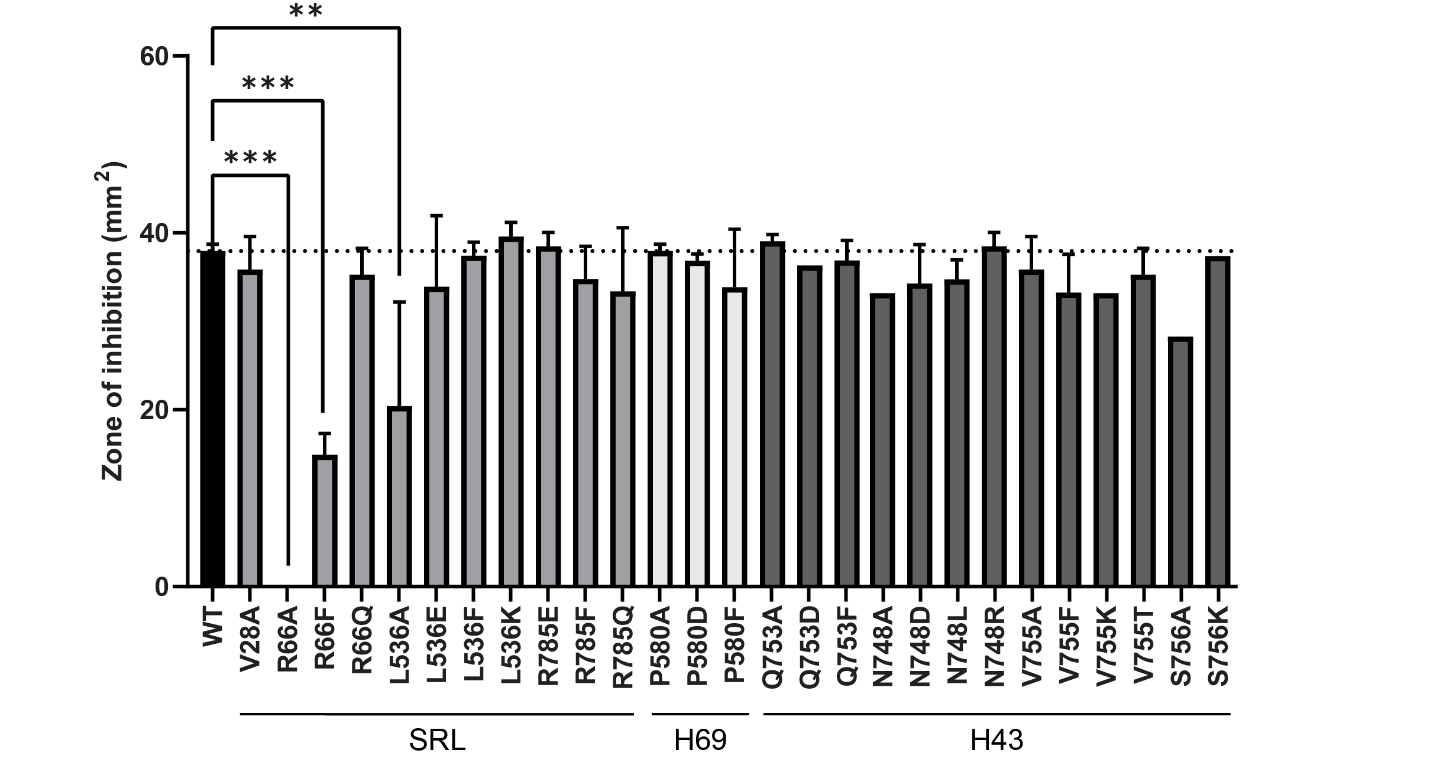


**Supplementary Figure 1.** Quantification of “Killer” data shown in Fig. 4C. Error bars denote standard deviation as calculated using students t-test (n=4). **denotes *p* <0.01, and ***denotes *p* < 0.001.
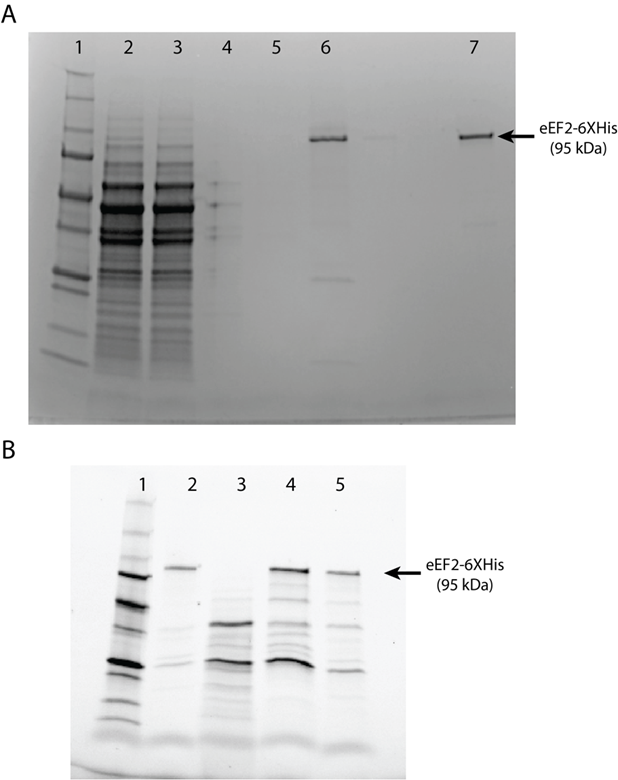


10

15

20

25

40

1200

190

50

60

85

**kDa**

**kDa**

60

40

1200

10

15

20

25

50

85

190

Supplementary Figure 2. Purification of eEF2-6XHis variants

A) Representative gel of eEF2 purification process, WT shown. Lanes: 1. Precision plus unstained protein standard (Bio-Rad), 2. Cell lysate, 3. Ni-NTA column flowthrough, 4. Ni-NTA column wash 1, 5. Ni-NTA column wash 2, 6. Ni-NTA purified protein, 7. Size-exclusion purified protein B) Ni-NTA purified eEF2 variants highlighting the lower MW contaminants that were present in several mutant strains and later removed through further purification. Relative amounts of eEF2-6XHis are not representative of final purified protein (representative example shown in 1A), only of their proportion to lower MW species. For example, R66A eEF2-6XHis appears absent, but was later observed after removal of contaminants (data not shown). Lanes: 1. Precision plus unstained protein standard, 2. WT, 3. R66A, 4. P580H, 5. Q753A.
